# Radiotherapy dose led to a substantial prolongation of survival in patients with locally advanced rectosigmoid junction cancer: a large population based study

**DOI:** 10.18632/oncotarget.8630

**Published:** 2016-04-07

**Authors:** Xu Guan, Zheng Jiang, Tianyi Ma, Zheng Liu, Hanqing Hu, Zhixun Zhao, Dawei Song, Yinggang Chen, Guiyu Wang, Xishan Wang

**Affiliations:** ^1^ Department of Colorectal Surgery, The Second Affiliated Hospital of Harbin Medical University, Harbin, China; ^2^ Department of Colorectal Surgery, Cancer Institute & Hospital, Chinese Academy of Medical Sciences, Peking Union Medical College, Beijing, China

**Keywords:** rectosigmoid junction cancer, radiotherapy, survival

## Abstract

Radiotherapy is widely applied for locally advanced rectal cancer (RC) to improve both local control and long-term outcomes. However, the efficacy of radiotherapy for rectosigmoid junction cancer (RSC) is still undetermined. Here, we identified 10074 patients who were diagnosed with locally advanced RSC from Surveillance, Epidemiology, and End-Results (SEER) cancer registry. These patients were divided into three subgroups according to different therapy strategies, including surgery alone, surgery plus preoperative radiotherapy and surgery plus postoperative radiotherapy. 5-year cancer-specific survival (CSS) and 5-year overall survival (OS) were obtained. Kaplan–Meier methods and Cox regression models were used to estimate the correlations between prognostic factors and survival outcomes. The 5-year CSSs for RSC patients treated with pre- and postoperative radiotherapy were 72.3% and 72.2%, which were significantly higher than surgery alone (64.8%). The 5-year OSs for RSC patients treated with pre- and postoperative radiotherapy were 71.6% and 71.2%, which were higher than surgery alone (64.0%). In the separate analyses of stage II and III RSC patients, the similar trends were also obtained. In addition, pre- and postoperative radiotherapy were equally identified as valuable prognostic factors for better survival outcomes in RSC patients. Furthermore, the results following propensity score matching also confirmed that the long-term survivals of RSC patients were improved following radiotherapy. In conclusion, locally advanced RSCpatients could obtain potential long-term survival benefits from radiotherapy. A prospective randomized control trial should be performed to further validate the strength of evidence in current study.

## INTRODUCTION

Colorectal cancer was the third most common cancer for both men and women over the world [1]. Currently, there has been obvious improvement for the outcome of patients with colorectal cancer due to the refinement of therapy modalities. Radiotherapy has been widely used to improve both local control and long-term survival for patients with locally advanced rectal cancer (RC), especially for the lower and middle RC [2, 3]. On the contrary, substantial evidences have suggested that radiotherapy is not beneficial to improve the outcomes for patients with colon cancer (CC). Therefore, radical colectomy without radiotherapy was generally recommended to treat patients with CC [4].

Although rectosigmoid junction was anatomically deemed as distal segment of sigmoid colon, some argued that it should be one part of rectum in consideration that it shared with crucial vascular system with the rectum above peritoneal reflection [5]. In fact, International Classification of Diseases for Oncology, Third Edition (ICD-O-3) recently noted that the rectosigmoid junction should be classified as one independent segment of large intestine (ICD-O; C-19), instead of categorizing it as colon (ICD-O; C-18) or rectum (ICD-O; C-20). The cancers at the rectosigmoid junction (RSC) should be therapeutically different from both CC and RC regarding to its special location. Actually, the role of pre- or postoperative radiotherapy in treatment of RSC still remains in suspense.

To our best knowledge, there is no available large population based evidence to confirm the effect of radiotherapy on locally advanced RSC based on the long-term outcomes. To address this issue, we assessed the long-term efficacy of radiotherapy for RSC according to different therapeutic strategies. Moreover, we also conducted a large-scale retrospective study to investigate the prognostic value of radiotherapy for sigmoid colon cancer (SC) and RC.

## RESULTS

### Patient characteristics

A total of 53932 patients were identified from the SEER database, including 24266 SC patients, 10074 RSC patients and 19592 RC patients. All demographics and pathological characteristics were summarized in Table 1. The proportion of younger patients who aged<60 increased gradually from 36.2% to 44.4% with the downward of tumor position. Male patients, white patients and patients in stage III accounted for larger proportions. As for histological type and tumor grade, the proportions of adenocarcinoma and grade II were respectively up to 92.0% and 73.8%. There was no significant difference of T stage and N stage among patients in three subgroups. 42.0% of RSC patients was presented with tumor size more than 5cm, the proportion was higher than SC (37.8%) and RC (34.0%). In addition, 41.0% of RC patients examined regional lymph node <12, which was obviously higher than SC (30.2%) and RSC (28.5%) patients.

**Table 1:** Comparison of clinical characteristics among patients with SC, RSC and RC

Characteristics	Sigmoid colon cancer	Rectosigmoid colon cancer	Rectal cancer	All patients
	N=24266 (%)	N=10074 (%)	N=19592 (%)	N=53932 (%)
**Age (Years)**				
**<60**	8793 (36.2)	4003 (39.7)	8707 (44.4)	21503 (39.9)
**≥60**	15473 (63.8)	6071 (60.3)	10885 (55.6)	32429 (60.1)
**Gender**				
**Male**	12739 (52.5)	5624 (55.8)	11703 (59.7)	30066 (55.7)
**Female**	11527 (47.5)	4450 (44.2)	7889 (40.3)	23866 (44.3)
**Race**				
**Black**	2537 (10.5)	926 (9.2)	1599 (8.2)	5062 (9.4)
**White**	18860 (77.7)	8058 (80.0)	15945 (81.4)	42863 (79.5)
**Other race**	2775 (11.4)	1054 (10.4)	2000 (10.2)	5829 (10.8)
**Unknown**	94 (0.4)	36 (0.4)	48 (0.2)	178 (0.3)
**AJCC Stage**				
**Stage II**	11350 (46.8)	4337 (43.1)	8353 (42.6)	24040 (44.6)
**Stage III**	12916 (53.2)	5737 (56.9)	11239 (57.4)	29892 (55.4)
**AJCC T stage**				
**T1/T2**	2244 (9.2)	1000 (9.9)	2228 (11.4)	5472 (10.1)
**T3/T4**	22022 (90.8)	9074 (90.1)	17364 (88.6)	48460 (89.9)
**AJCC N stage**				
**N0**	11350 (46.8)	4337 (43.1)	8353 (42.6)	24040 (44.6)
**N1/N2**	12916 (53.2)	5737 (56.9)	11239 (57.4)	29892 (55.4)
**Histological type**				
**Adenocarcinoma**	22496 (92.7)	9344 (92.8)	17780 (90.8)	49620 (92.0)
**Mucinous/Signet- ring cell cancer**	1678 (6.9)	690 (6.8)	1598 (8.2)	3966 (7.4)
**Others**	92 (0.4)	40 (0.4)	214 (1.0)	346 (0.6)
**Grade**				
**Grade I**	1700 (7.0)	600 (6.0)	1175 (6.0)	3475 (6.4)
**Grade II**	18503 (76.3)	7542 (74.9)	13734 (70.1)	39779 (73.8)
**Grade III**	3263 (13.4)	1494 (14.8)	2883 (14.7)	7640 (14.2)
**Grade IV**	316 (1.3)	153 (1.5)	263 (1.4)	732 (1.3)
**Unknown**	484 (2.0)	285 (2.8)	1537 (7.8)	2306 (4.3)
**The number of lymph nodes**				
**< 12**	7336 (30.2)	2873 (28.5)	8038 (41.0)	18247 (33.8)
**≥ 12**	16748 (69.0)	7121 (70.7)	11309 (57.7)	35178 (65.2)
**Unknown**	182 (0.8)	80 (0.8)	245 (1.3)	507 (1.0)
**Tumor size (cm)**				
**< 2**	1135 (4.7)	415 (4.1)	1335 (6.8)	2885 (5.4)
**2-5**	12565 (51.8)	4702 (46.7)	8318 (42.5)	25585 (47.4)
**≥ 5**	9175 (37.8)	4228 (42.0)	6666 (34.0)	20069 (37.2)
**Unknown**	1391 (5.7)	729 (7.2)	3273 (16.7)	5393 (10.0)
**Therapy**				
**Preoperative radiotherapy**	136 (0.6)	1194 (11.8)	10977 (56.0)	12307 (22.8)
**Postoperative radiotherapy**	865 (3.5)	1992 (19.8)	4026 (20.6)	6883 (12.8)
**Surgery alone**	23265 (95.9)	6888 (68.4)	4589 (23.4)	34742 (64.4)

Other race (American Indian/AK Native, Asian/Pacific Islander).

**Figure 1:**
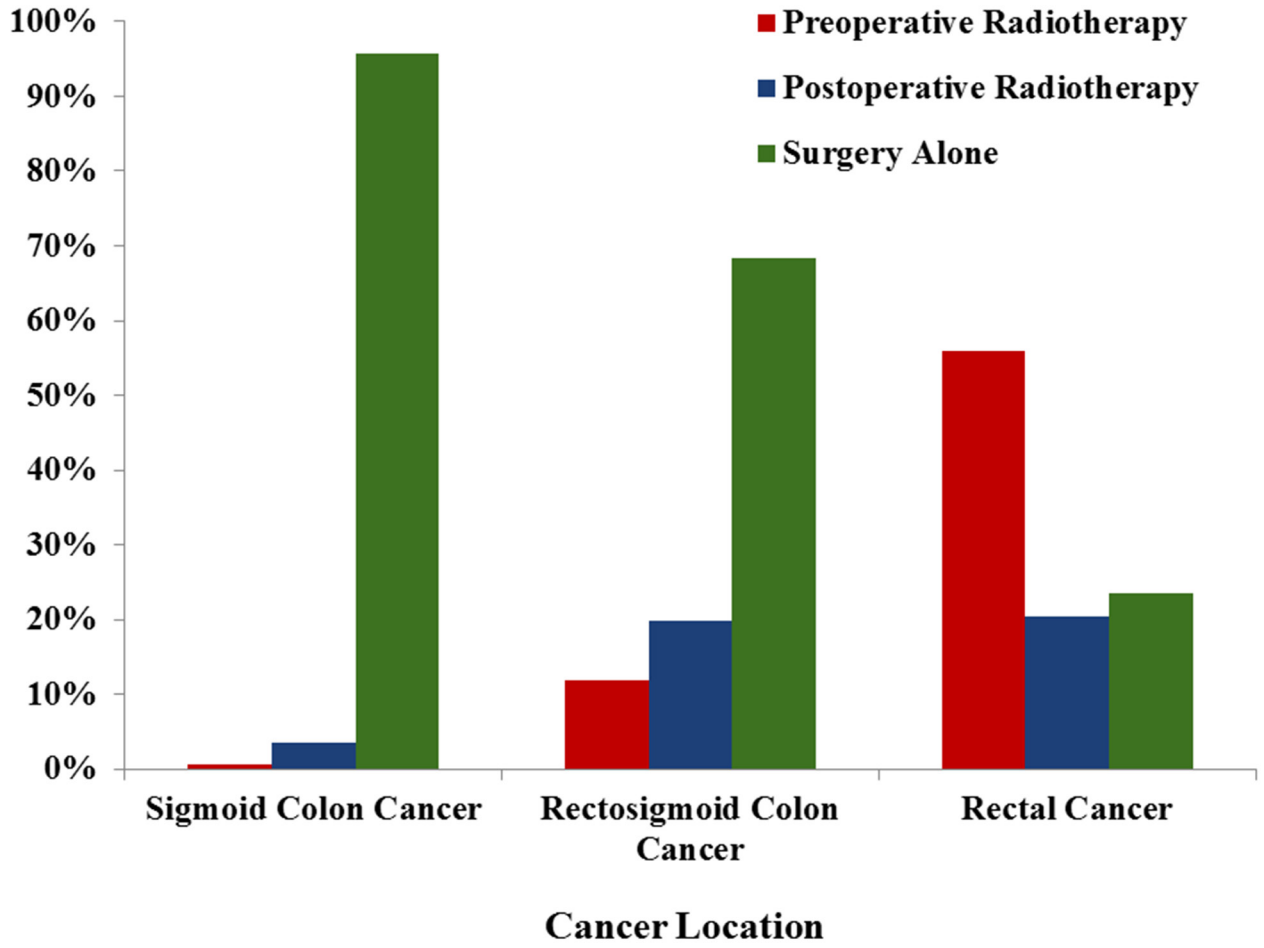
Patient proportions according to different therapeutic strategies

The proportion of patients receiving preoperative radiotherapy was varied with tumor locations. For RC patients, 56.0% of patients received preoperative radiotherapy, which was obviously higher than those patients with SC (0.6%) and RSC (11.8%). The proportion of surgery without radiotherapy was 95.9% in SC patients, 68.4% in RSC patients and 23.4% in RC patients (Figure 1).

### Comparisons of CSS for patients with SC, RSC and RC

**Figure 2:**
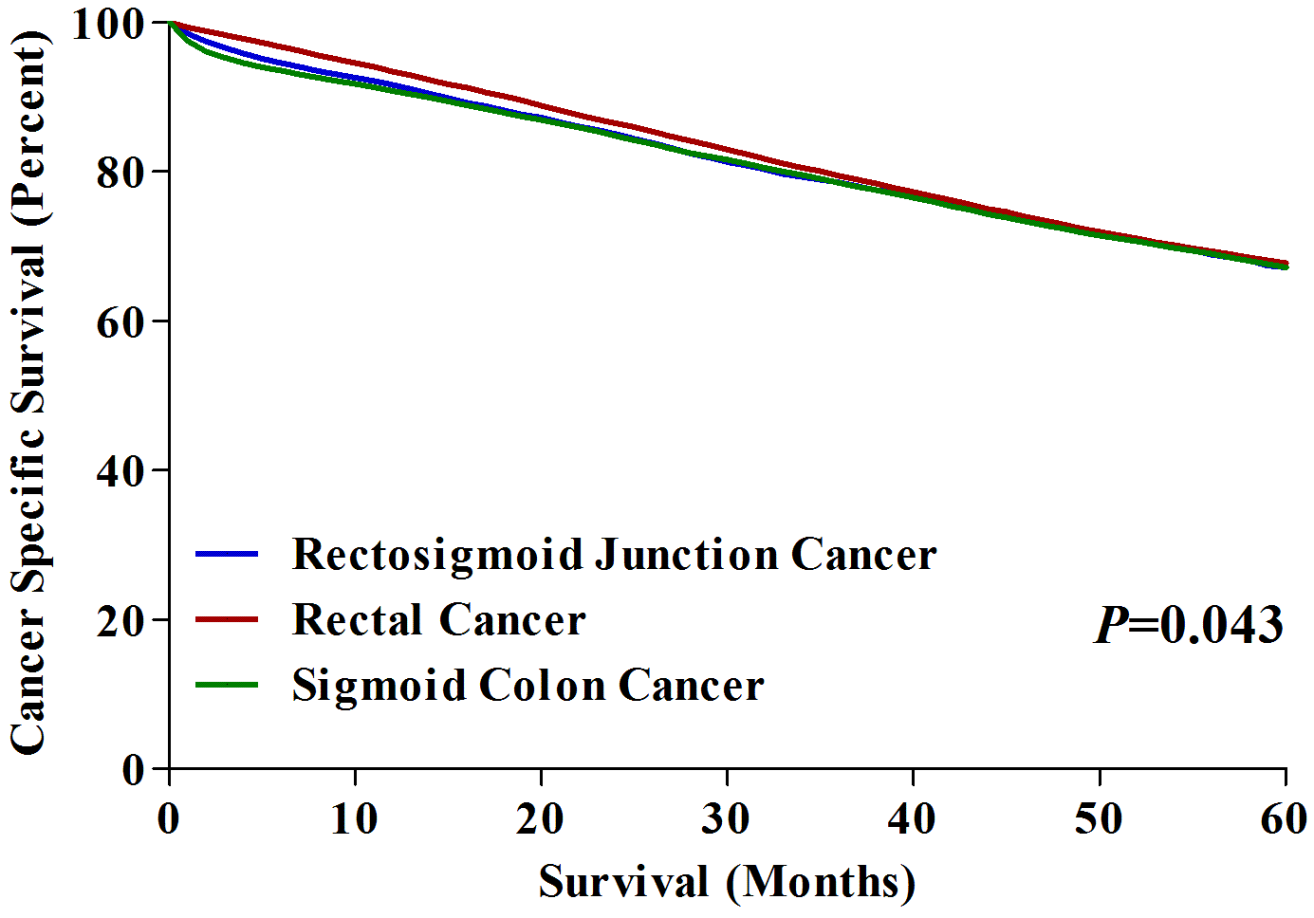
5-year CSSs in patients with SC, RSC and RC

The 5-year CSS of RSC patients was 67.2%, which was same to that of SC patients (67.2%) and lower than that of RC patients (67.8%). We made an overall comparison of CSSs for patients with SC, RCS and RC, which showed significant difference among three groups (P=0.043) (Figure 2). Furthermore, we performed pairwise comparisons of CSSs in three groups respectively. The results showed that there was significant difference on CSS between RC and SC patients (P=0.013), but no difference on CSSs between RC and RSC patients (P=0.160), nor between SC patients and RSC patients (P=0.572).

### Comparisons of long-term outcomes based on different therapy strategies

**Figure 3:**
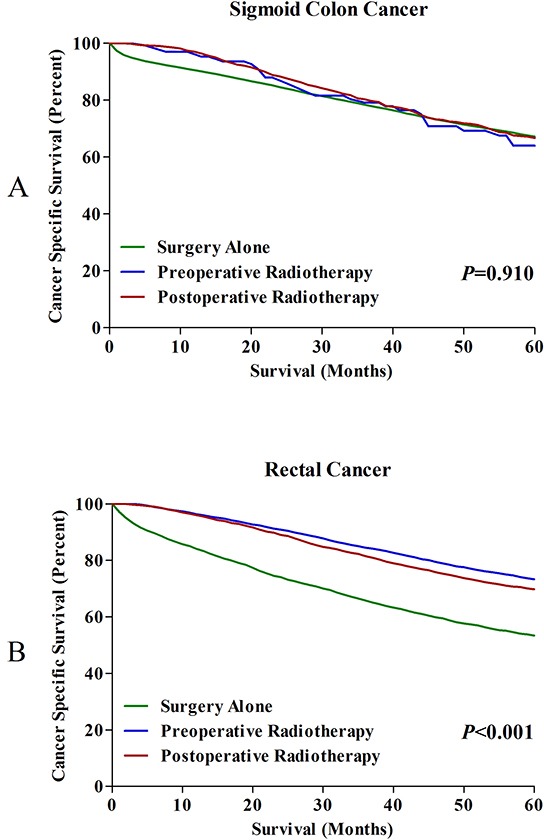
**A.** 5-year CSSs in SC patients treated with surgery alone, pre- and postoperative radiotherapy. **B.** 5-year CSSs in RC patients treated with surgery alone, pre- and postoperative radiotherapy.

With the aim of comparing the long-term outcomes for patients with SC, RSC and RC, we divided each of three groups into three subgroups based on different therapy strategies. For SC patients, we found that there was no significant difference on CSS among three subgroups (P=0.910), which indicated that radiotherapy could not extend the 5-year CSS for SC patients (Figure 3A). In RC group, we found that the 5-year CSSs were respectively 73.3%, 69.8% and 53.4% for patients treated with pre-, post-operative radiotherapy and surgery alone. The CSSs among these subgroups was significantly different, with P<0.001. Interestingly, the 5-year CSS was also significantly different between pre- and post-operative radiotherapy (P<0.001) (Figure 3B).

**Figure 4:**
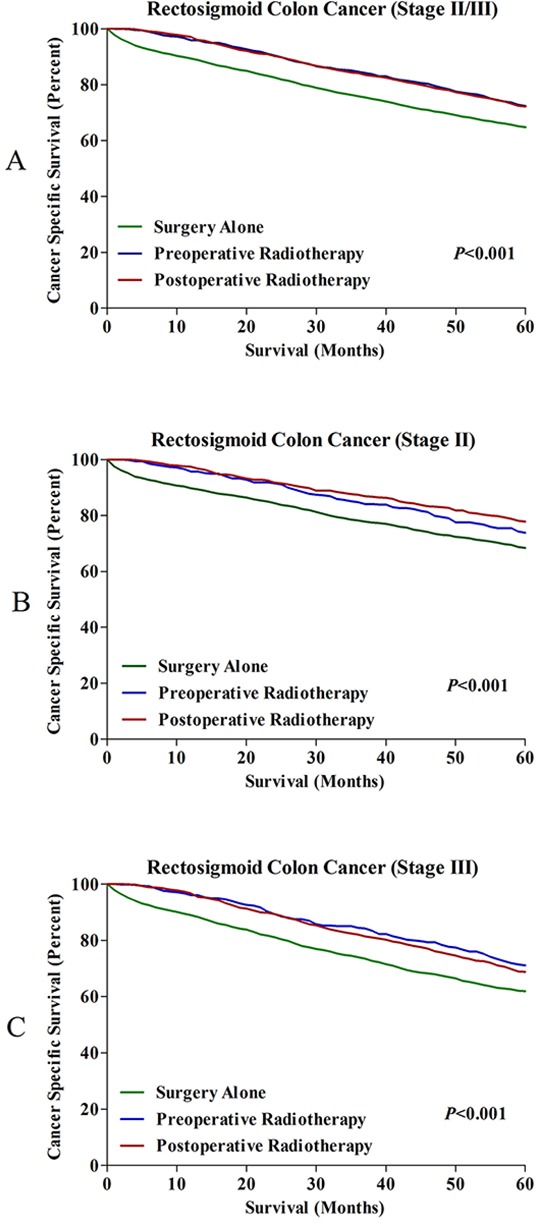
**A.** 5-year CSSs in all RSC patients treated with surgery alone, pre- and postoperative radiotherapy. **B.** 5-year CSSs in stage II RSC patients treated with surgery alone, pre- and postoperative radiotherapy. **C.** 5-year CSSs in stage III RSC patients treated with surgery alone, pre- and postoperative radiotherapy.

For patients with RSC, the results showed that the 5-year CSSs for patients treated with pre- and post-operative radiotherapy were 72.3% and 72.2%, which was significantly higher than those received surgery alone (64.8%) (Figure 4A). Furthermore, we analyzed the effects of radiotherapy on CSSs for stage II (Figure 4B) and stage III (Figure 4C) RSC patients, respectively. The results showed that both of them could gain survival benefits from pre- or post-operative radiotherapy compared to surgery alone. In addition, the OSs of RSC patients were also compared, we found that the 5-year OSs for RSC patients treated with pre- and post-operative radiotherapy were 71.6% and 71.2%, the 5-year OSs for RSC patients who underwent surgery alone was only 64.0%, which indicated the OS benefit from radiotherapy remained unchanged for RSC patients (Supplementary Figure S1A). The OS comparisons in stage II (Supplementary Figure S1B) and stage III (Supplementary Figure S1C) RSC patients could also obtained the similar results.

### Identification of prognostic factors for patients with SC, RSC and RC

To further explore the prognostic factors for patients with SC, RSC and RC, we performed exploratory analyses to identify patient-, tumor-, and treatment-associated prognostic factors. Univariate and multivariate regression analyses were used to determine prognostic factors in three groups of patients separately. Characteristics including age ≥60, black, stage III, mucinous/signet ring cell cancer and grade III/IV were identified as independent poor prognostic factors for CSS in patients with SC, RSC and RC (Table 2–4). However, as for the treatment factors, the results were inconsistent among three groups. For SC patient, there was no significant difference based on different therapy strategies (P=0.279) (Table 2), which indicated that radiotherapy couldn't impact survival outcome for SC patients. For RSC patients, we found that pre- and post-operative radiotherapy were equally identified as significant prognostic factors for better survival outcomes (Table 3). For RC patients, the preoperative radiotherapy had the lowest HR for CSS (Table 4), which implied that preoperative radiotherapy could be considered as a prognostic factor to improve long-term survival.

**Table 2:** Univariate and multivariate analyses for SC patients

Characteristic		Univariate analysis		Multivariate analysis	
		HR [95% CI]	P	HR [95% CI]	P
**Gender**	Female	1	0.041	1	0.194
	Male	1.050 [1.002-1.101]		1.032 [0.984-1.082]	
**Age**	<60	1	<0.001	1	<0.001
	≥60	2.562 [2.415-2.718]		2.665 [2.511-2.829]	
**Race**	White	1	<0.001	1	<0.001
	Black	1.139 [1.058-1.227]	0.001	1.256 [1.166-1.354]	<0.001
	Other race	0.745 [0.685-0.810]	<0.001	0.781 [0.718-0.850]	<0.001
**AJCC Stage**	Stage II	1	<0.001	1	<0.001
	Stage III	1.162 [1.108-1.219]		1.254 [1.195-1.316]	
**Histological type**	Adenocarcinoma	1	<0.001	1	<0.001
	Mucinous/Signet ring cell cancer	1.438 [1.327-1.558]	<0.001	1.354 [1.248-1.469]	<0.001
	Others	2.139 [1.568-2.919]	<0.001	1.775 [1.298-2.427]	<0.001
**Grade**	Grade I	1	<0.001	1	<0.001
	Grade II	0.962 [0.876-1.057]	0.419	1.000 [0.910-1.100]	0.994
	Grade III	1.370 [1.232-1.523]	<0.001	1.393 [1.252-1.550]	<0.001
	Grade IV	1.537 [1.243-1.900]	<0.001	1.584 [1.281-1.960]	<0.001
**Therapy**	Surgery alone	1	0.910	1	0.279
	Preoperative radiotherapy	1.045 [0.760-1.438]	0.785	1.200 [0.872-1.652]	0.262
	Postoperative radiotherapy	0.979 [0.865-1.109]	0.739	1.076 [0.950-1.218]	0.248

Other race (American Indian/AK Native, Asian/Pacific Islander).

**Table 3:** Univariate and multivariate analyses for RSC patients

Characteristic		Univariate analysis		Multivariate analysis	
		HR [95% CI]	P	HR [95% CI]	P
**Gender**	Female	1	0.036	1	0.001
	Male	1.083 [1.005-1.166]		1.135 [1.054-1.223]	
**Age**	<60	1	<0.001	1	<0.001
	≥60	2.525 [2.313-2.756]		2.536 [2.321-2.772]	
**Race**	White	1	<0.001	1	<0.001
	Black	1.281 [1.139-1.441]	<0.001	1.388 [1.233-1.561]	<0.001
	Other race	0.722 [0.628-0.830]	<0.001	0.748 [0.651-0.860]	<0.001
**AJCC Stage**	Stage II	1	<0.001	1	<0.001
	Stage III	1.226 [1.137-1.322]		1.285 [1.191-1.387]	
**Histological type**	Adenocarcinoma	1	<0.001	1	<0.001
	Mucinous/Signet ring cell cancer	1.669 [1.478-1.885]	<0.001	1.596 [1.412-1.804]	<0.001
	Others	1.733 [1.060-2.833]	0.028	1.698 [1.036-2.781]	0.036
**Grade**	Grade I	1	<0.001	1	<0.001
	Grade II	1.215 [1.028-1.436]	0.022	1.265 [1.071-1.496]	0.006
	Grade III	1.723 [1.436-2.068]	<0.001	1.697 [1.413-2.038]	<0.001
	Grade IV	1.879 [1.349-2.617]	<0.001	1.925 [1.381-2.683]	<0.001
**Therapy**	Surgery alone	1	<0.001	1	<0.001
	Preoperative radiotherapy	0.692 [0.607-0.788]	<0.001	0.763 [0.669-0.871]	<0.001
	Postoperative radiotherapy	0.693 [0.628-0.764]	<0.001	0.741 [0.671-0.818]	<0.001

Other race (American Indian/AK Native, Asian/Pacific Islander).

**Table 4:** Univariate and multivariate analyses for RC patients

Characteristic		Univariate analysis		Multivariate analysis	
		HR [95% CI]	P	HR [95% CI]	P
**Gender**	Female	1	0.059	1	0.245
	Male	0.948 [0.898-1.002]		1.033 [0.978-1.092]	
**Age**	<60	1	<0.001	1	<0.001
	≥60	2.176 [2.050-2.309]		2.046 [1.926-2.173]	
**Race**	White	1	<0.001	1	<0.001
	Black	1.256 [1.146-1.376]	<0.001	1.286 [1.174-1.410]	<0.001
	Other race	0.850 [0.772-0.936]	0.001	0.884 [0.802-0.973]	0.012
**AJCC Stage**	Stage II	1	<0.001	1	<0.001
	Stage III	1.286 [1.216-1.359]		1.338 [1.264-1.416]	
**Histological type**	Adenocarcinoma	1	<0.001	1	<0.001
	Mucinous/Signet ring cell cancer	1.473 [1.353-1.604]	<0.001	1.425 [1.307-1.553]	<0.001
	Others	1.398 [1.111-1.759]	0.004	1.333 [1.057-1.682]	0.015
**Grade**	Grade I	1	<0.001	1	<0.001
	Grade II	1.023 [0.909-1.153]	0.703	0.994 [0.882-1.120]	0.920
	Grade III	1.589 [1.397-1.808]	<0.001	1.472 [1.293-1.675]	<0.001
	Grade IV	2.181 [1.742-2.732]	<0.001	1.912 [1.524-2.398]	<0.001
**Therapy**	Surgery only	1	<0.001	1	<0.001
	Preoperative radiotherapy	0.446 [0.419-0.474]	<0.001	0.503 [0.472-0.536]	<0.001
	Postoperative radiotherapy	0.546 [0.508-0.588]	<0.001	0.568 [0.528-0.612]	<0.001

Other race (American Indian/AK Native, Asian/Pacific Islander).

### Long term survival analyses following propensity score matching

After propensity score matching, there were totally 6372 patients left, with 1:1 ratio in surgery alone group and surgery plus radiotherapy group. The characteristics between two groups were well balance in the aspect of gender, race, AJCC stage, histology type and the number lymph nodes examined. Supplementary Table S1 showed the changes of all characteristics before and after propensity score matching. The 5-year CSSs for patients treated surgery plus radiotherapy were 72.2%, which was significantly higher than those received surgery alone (66.9%) (Supplementary Figure S2A). The 5-year OSs for patients treated radiotherapy were 71.9%, which was also higher than patients received surgery alone (66.5%) (Supplementary Figure S2B).

## DISCUSSION

Colorectal cancer is one heterogeneous disease, showing variety of epidemiological, pathological and genetic characteristics altered according to tumor locations [7–9], which may cause differences in prognosis and treatment strategy. RSC was currently considered as one independent cancer without being classified as SC or RC, and its optimal therapeutic strategy might be different from SC and RC. In this study, our results showed that the combination of surgery and radiotherapy was the preferred therapeutic strategy for RSC patients, which could markedly improve the 5-year CCS and OS compared with surgery alone. In addition, we also confirmed that preoperative radiotherapy followed by surgery could obviously improve long-term survival outcomes compared with surgery alone, but radiotherapy was not available to improve the prognosis of SC patients.

Accumulating evidence has supported that RSC should be considered as an independent cancer group, according to its particular association with a special anatomic localization, the high frequency of intestinal obstruction, and worse long-term outcome compared with upper rectal cancer [10]. However, the definition of rectosigmoid junction is a highly controversial issue. Some argued that it should be defined as the segment of large bowel between the sacral promontory and the lower margin of S2 [10]. In the SEER database, the definition of rectosigmoid junction colon is not elaborated in detail. Moreover, the database included hundreds of US hospitals which had their own criteria to define the tumor location. These two factors contributed to the heterogeneity in the analyses, which might lead to biased result. Specifically, some upper rectal cancer might be regarded as the rectosigmoid cancer according to the foregoing definition, which results in neutralizing the worsening trends in prognosis of patients underwent radiotherapy to some extent. Therefore, the conclusion of this article should be made with caution.

There was no large population based study to explore the optimal therapeutic strategy for RSC patients based on the long-term survival. Here, we found that there was no difference between preoperative and postoperative radiotherapy on 5-year CSS and OS of RSC patients, and both of treatment modalities were significantly better than surgery alone. In fact, we found that surgery without radiotherapy was widely accepted for the treatment of RSC patients in United States, showing 68.4% of RSC patients being managed with surgery alone, this proportion was significantly higher than patients who treated with radiotherapy plus surgery. Therefore, it may be reasonable to modify the therapeutic modality from surgery alone to the combination of surgery and radiotherapy for patients with locally advanced RSC.

Radiotherapy has contributed to marked improvement of outcomes in patients with locally advanced RC. Previous studies have shown that radiotherapy could effectively improve long-term outcomes and local control for RC patients [11–14]. Recently, a variety of clinical trials comparing the efficacy of preoperative radiotherapy with postoperative radiotherapy schedules were designed to optimize the sequence of therapeutic modalities in the treatment of locally advanced RC. Compared with postoperative radiotherapy, although preoperative radiotherapy obviously decreased local recurrence rate and increased sphincter preservation rate, the role on long-term outcome is still controversial [15–18]. In our study, we found that preoperative radiotherapy significantly improves 5-year CSS, which highlights the key role of preoperative radiotherapy in the treatment of locally advanced RC patients.

Although the strengths of this study including large sample size, propensity score matching test, many limitations should be explained. First of all, local recurrence would more likely be one primary endpoint in this study [15, 19–21], but the SEER lack the recurrence data which contribute to the local control benefit of radiotherapy for RSC couldn't be analyzed, instead of CSS and OS. Secondly, the SEER has no detailed information associated with treatment compliance, toxicity and histopathologic features including angiolymphatic invasion and margin of resection. All these factors are presented with prognostic value in colorectal cancer treatments. Finally, because of the limited number of RSC patients who received radiotherapy in our center, it was a pity that we cannot provide the true cases of RSC in this study.

In conclusion, both pre- and postoperative radiotherapy improve the long-term outcome for patients with locally advanced RSC, and the similar results were seen even after propensity score matching. However, further studies in prospective randomized trials are warranted and may prove the advantages of this procedure in the present study.

## MATERIALS AND METHODS

### Data resources

We extracted the data from the Surveillance, Epidemiology, and End-Results (SEER) cancer registry [6]. The SEER database covers 28% percent of the US population, including the information of demographic, incidence and survival data from 17 population-based cancer registries. The SEER is one openly accessed database, and data in SEER could be available for the research. Data extracted from this database do not need informed patient consent, because they were anonymized and de-identified prior to release. We have got permission to extract data file in the SEER program by National Cancer Institute, USA and the reference number was 11228-Nov2014.

### Study population

We collected the patients pathologically diagnosed with SC, RSC, and RC. All these patients were diagnosed between 2004 and 2012, because the 7^th^ edition of American Joint Committee on Cancer (AJCC) stage system was available in SEER since 2004. The collected patients were confined to locally advanced stage, which was defined as stage II (T3-4, node-negative disease with tumor penetration through muscle wall) or stage III (node-positive disease without distant metastasis) according to TNM stage system. The treatment strategies for these patients included surgery alone, surgery plus preoperative radiotherapy and surgery plus postoperative radiotherapy. In addition, other available characteristics included age, gender, race, AJCC stage, T stage, N stage, histological type, tumor size, the number of regional lymph nodes examined and tumor grade. The exclusion criteria included the patients: dead due to other causes, alive with no survival time and with distant metastasis.

### Statistical analysis

The primary endpoints of this study included cancer-specific survival (CSS) and overall survival (OS). The OS was defined as the time from cancer diagnosis until all causes of death or the end of follow up. The CSS was defined as the time from the cancer diagnosis until occurrence of cancer-related death or the end of follow up. Deaths caused by other reasons were considered as censored cases. The CSS and OS were estimated using Kaplan-Meier method, and log-rank test was used to compare the differences of CSS and OS respectively. Univariate and multivariate Cox's regression models were also performed to estimate hazard rate (HR) and exact 95% confidence intervals (CIs). All statistical tests were two sided, P<0.05 was considered to be statistical significance. All statistical analyses were performed by using SPSS statistical software, version 20 (IBM Corp, Armonk, NY, USA) and R version 2.12.0 (www.r-project.org).

### Propensity score matching

A propensity 1:1 matched analysis was done to reduce possible bias to a minimum in this retrospective analysis. Propensity scores were calculated using logistic regression model for each patient in both surgery alone group and surgery plus radiotherapy group. The covariates included in the regression were age, gender, race, AJCC stage, histological type, grade, the number of lymph nodes examined, and tumor size. Patients in two groups were matched based on the propensity score. Covariates balance between two groups was examined by χ^2^ test. The CSS and OS analyses were then performed for the propensity score-matched patients using the same methods as those in the primary analysis.

## SUPPLEMENTARY FIGURES AND TABLES


