# Supplementary material for: Radiotherapy dose led to a substantial prolongation of survival in patients with locally advanced rectosigmoid junction cancer: a large population based study
Source: Oncotarget. 2016 Apr 7;7(19):28408–19. doi: 10.18632/oncotarget.8630 (PMC5053735; doi:10.18632/oncotarget.8630)
Supplement: Supplementary file 1 [file oncotarget-07-28408-s001.pdf]

SUPPLEMENTARY FIGURES AND TABLE

A

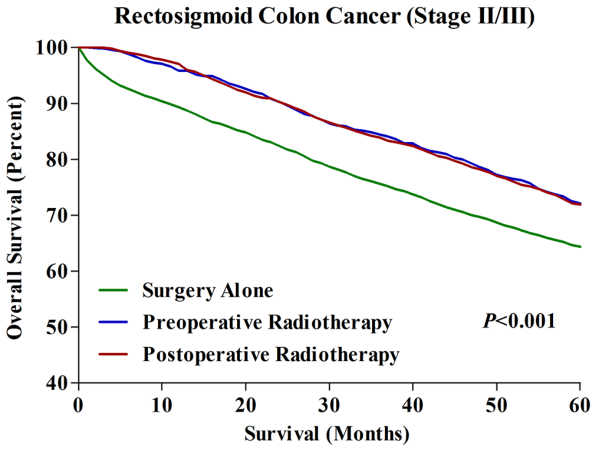

B

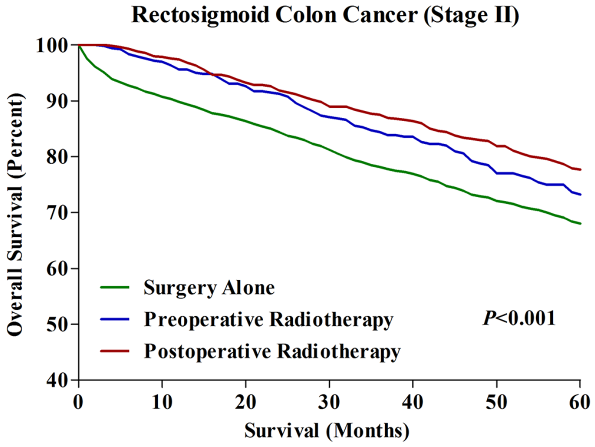

C

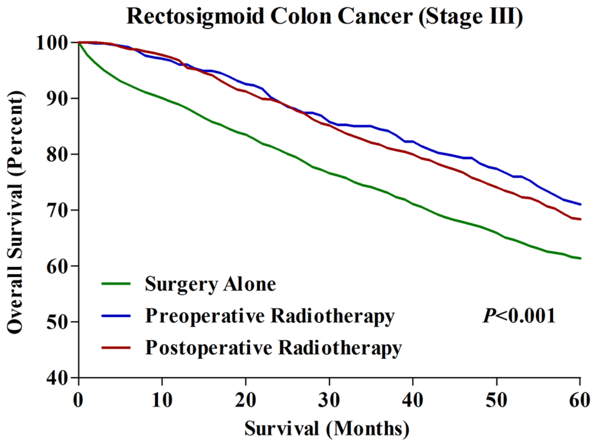

Supplementary Figure S1: A. 5-year OSs in all RSC patients treated with surgery alone, pre- and postoperative radiotherapy. **B.** 5-year OSs in stage II RSC patients treated with surgery alone, pre- and postoperative radiotherapy. **C.** 5-year OSs in stage III RSC patients treated with surgery alone, pre- and postoperative radiotherapy.

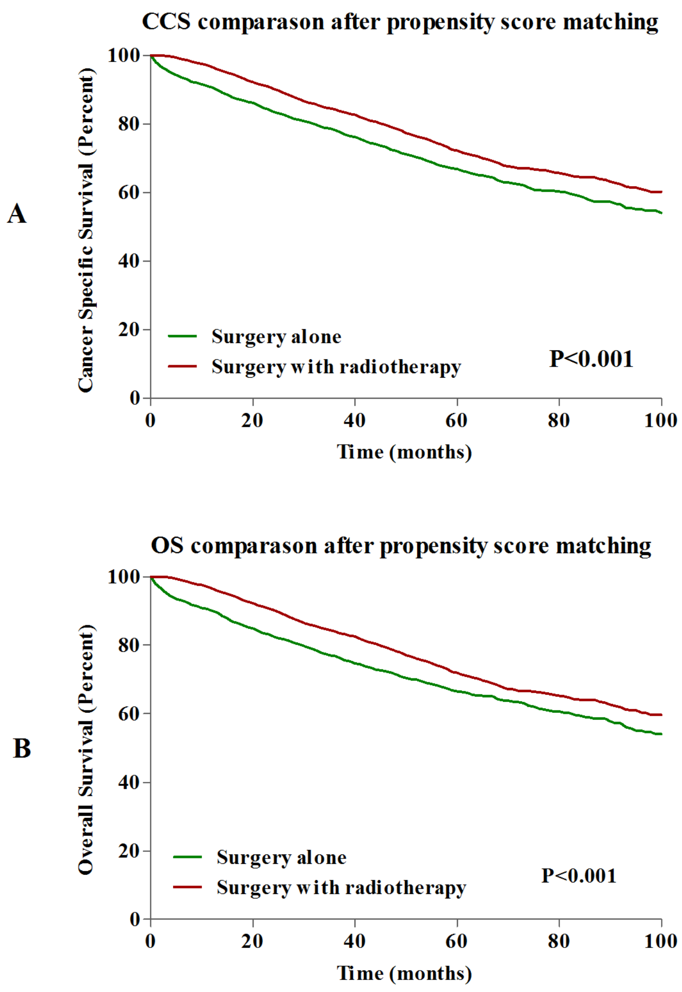

Supplementary Figure S2: A. Comparisons of 5-year CSS after propensity score matching. **B.** Comparisons of 5-year OS after propensity score matching.

Supplementary Table S1: Characteristics among patients with or without radiotherapy before and after propensity score matching

Characteristics	Before matching			After matching		
	Surgery alone	Surgery with radiotherapy		Surgery alone	Surgery with radiotherapy	
Age (Years)			<0.001			0.005
<60	2403 (34.9)	1600 (50.2)		1130 (46.5)	1600 (50.2)	
≥60	4485 (65.1)	1586 (49.8)		1302 (53.5)	1586 (49.8)	
Gender			<0.001			0.517
Male	3692 (53.6)	1932 (60.6)		1454 (59.8)	1932 (60.6)	
Female	3196 (46.4)	1254 (39.4)		978 (40.2)	1254 (39.4)	
Race			0.450			0.978
Black	640 (9.3)	286 (9.0)		211 (8.7)	286 (9.0)	
White	5525 (80.2)	2533 (79.5)		1944 (79.9)	2533 (79.5)	
Other race	698 (10.1)	356 (11.2)		269 (11.1)	356 (11.2)	
Unknown	25 (0.4)	11 (0.3)		8 (0.3)	11 (0.3)	
AJCC Stage			0.001			0.924
Stage II	3040 (44.1)	1297 (40.7)		987 (40.6)	1297 (40.7)	
Stage III	3848 (55.9)	1889 (59.3)		1445 (59.4)	1889 (59.3)	
Histological type			0.025			0.113
Adenocarcinoma	6421 (93.2)	2923 (91.7)		2256 (92.8)	2923 (91.7)	
Mucinous/Signet-ring cell cancer	443 (6.4)	247 (7.8)		171 (7.0)	247 (7.8)	
Others	24 (0.3)	16 (0.5)		5 (0.2)	16 (0.5)	
Grade			<0.001			0.026
Grade I	418 (6.1)	182 (5.7)		148 (6.1)	182 (5.7)	
Grade II	5225 (75.9)	2317 (72.7)		1798 (73.9)	2317 (72.7)	
Grade III	1009 (14.6)	485 (15.2)		379 (15.6)	485 (15.2)	
Grade IV	103 (1.5)	50 (1.6)		32 (1.3)	50 (1.6)	
Unknown	133 (1.9)	152 (4.8)		75 (3.1)	152 (4.8)	
The number of lymph nodes			<0.001			0.591
< 12	1846 (26.8)	1027 (32.2)		989 (31.1)	1027 (32.2)	
≥ 12	4990 (72.4)	2131 (66.9)		2169 (68.0)	2131 (66.9)	
Unknown	52 (0.8)	28 (0.9)		28 (0.9)	28 (0.9)	
Tumor size (cm)			<0.001			0.037
< 2	242 (3.5)	173 (5.4)		127 (4.0)	173 (5.4)	
2-5	3395 (49.3)	1307 (41.0)		1367 (42.9)	1307 (41.0)	
≥ 5	2871 (41.7)	1357 (42.6)		1351 (42.4)	1357 (42.6)	
Unknown	380 (5.5)	349 (11.0)		341 (10.7)	349 (11.0)	
